# Virucidal activity of chlorine dioxide in combination with acetic acid or citric acid and a surfactant, in presence of interfering substances, against polio-, adeno- and murine norovirus in suspension-, carrier- and four-field tests

**DOI:** 10.3205/dgkh000492

**Published:** 2024-08-12

**Authors:** Patryk Tarka, Arkadiusz Chruściel, Wiesław Hreczuch, Krzysztof Kanecki, Aneta Nitsch-Osuch

**Affiliations:** 1 Department of Social Medicine and Public Health, Medical University of Warsaw, Warsaw, Poland; 2 MEXEO, Kedzierzyn-Kozle, Poland

**Keywords:** chlorine dioxide, acetic acid, citric acid, virucidal activity, EN 14476, EN 16777, EN 16615, norovirus, adenovirus, poliovirus

## Abstract

**Introduction:**

The aim of the study was to investigate whether the virucidal effectiveness of chlorine dioxid against adenovirus and murine norovirus can be improved by combining it with carboxylic acids and surfactants.

**Method:**

The virucidal efficacy against polio-, adeno- and murine norovirus has been tested in presence of interfering substances in the quantitative suspension test according to EN 14476, the carrier test without mechanical action according to EN 16777, and in the four-field test according to EN 16615.

Three chlorine-dioxide-based surface disinfectants were tested: a two-component cleaning disinfectant concentrate for large surfaces, a ready-to-use (RTU) foam, and an RTU gel.

**Results:**

Cleaning and disinfecting preparations based on chlorine dioxide, applied at various concentrations, in combination with acetic acid or citric acid and surfactants, are virucidally active against polio-, adeno-, and norovirus after an exposure time of 5 minutes in presence of interfering substances.

## Zusammenfassung

**Einleitung:**

Ziel der Studie war es, zu untersuchen, ob die viruzide Wirksamkeit von Hypochlorit gegen Adenovirus und murines Norovirus durch die Kombination mit Carbonsäuren und Tensiden verbessert werden kann.

**Methode:**

Die viruzide Wirksamkeit wurde gegen Polio-, Adeno- und Noroviren jeweils unter hoher Belastung im quantitativen Suspensionstest nach EN 14476, im quantitativen Keimträgertest ohne mechanische Einwirkung nach EN 16777 und im Vierfeldertest nach EN 16615 geprüft.

Es wurden drei oberflächenaktive Flächendesinfektionsmittel auf Basis von Chlordioxid getestet: ein Zweikomponenten Reinigungs-Desinfektionsmittel-Konzentrat für große Flächen, ein gebrauchsfertiger Schaum und ein gebrauchsfertiges Gel.

**Ergebnisse:**

Die geprüften Desinfektionsmittel auf Basis von Chlordioxid in Kombination mit Essigsäure oder Zitronensäure und einem Tensid zeigten bei verschiedenen Chlordioxidkonzentrationen nach 5 min Einwirkungszeit unter hoher Belastung eine viruzide Wirksamkeit gegen Polio-, Adeno- und Noroviren.

## Introduction

There is no vaccine or specific antiviral therapy available to prevent or treat human norovirus (HuNoV) infections [1]. Since norovirus can be transmitted through contaminated surfaces, disinfection is particularly important [2], [3], [4], [5]. Sodium hypochlorite (chlorine bleach) is advised by the CDC and ECDC as the gold standard disinfectant for use in norovirus outbreaks [6], [7]. According to the CDC, the required concentration for cleaning environmental surfaces is between 1,000 and 5,000 ppm [6], [7]. A concentration of 1,000 ppm chlorine was shown to have limited effectiveness on surfaces that are notably contaminated with biological material [8]. Therefore, cleaning with a detergent and warm water prior to using bleach is required to remove all organic matter. The use of sodium hypochlorite at such a high concentration is unacceptable in many locations due to its corrosivity and/or potential damage to many surfaces, strong and irritating odor, potential hazard to user health and the environment, and reduced effectiveness in the presence of organic material [9], [10]. Furthermore, sodium hypochlorite preparations are associated with risks from their by-products, which may pose a threat to humans and the environment [11]. Given the limitations of sodium-hypochlorite-based products, alternative disinfectants are needed with improved norovirus inactivation and more favorable safety and material compatibility profiles. 

In this context, chlorine dioxide is of great interest, because its virucidal activity is 10-fold stronger than that of sodium hypochlorite, without the previously mentioned disadvantages of chlorine compounds [11], [12]. Chlorine dioxide is an inert chlorine compound. It differs from elemental chlorine both in terms of chemical structure and behavior [13]. Because of rapid decomposition, its oxidizing effect on the skin is milder than that of sodium hypochlorite, even at very high concentrations [14]. Moreover, chlorine dioxide is a very strong oxidant capable of destroying bacteria, fungi, viruses, spores, and protozoa [15], [16], [17], [18], [19], [20], [21], [22], [23], [24]. It is used in toothpastes and mouthrinses [25], [26]. The aim of the study was to test the virucidal activity of three chlorine-dioxide–based disinfectants against poliovirus, adenovirus, and murine norovirus.

Difficulties in culturing HuNoV have resulted in the search for other model viruses (surrogates) of norovirus that are easier to cultivate [27], [28]. Successful HuNoV cultivation in stem-cell-derived human intestinal enteroids (HIEs) has recently been reported [29]. Owing to the cost of commercial media, the time required to run the assays, and the lack of quantifiability, the use of the HIE model for the broad screening of product efficacy is impractical [29]. In particular, the lack of quantifiability is one of the major limitations of the current HIE model for HuNoV [29]. Traditional methods used to assess the virucidal effect of disinfectants are based on cell cultures and are considered the gold standard. Therefore, in 2011, the CEN (European Committee for Standardization) additionally introduced the murine norovirus (MNV) as a test virus into the EN 14476 [30]. It is an easily cultured HuNoV surrogate, and, at the same time, it achieves high titers and has a favorable safety profile for laboratory personnel [28], [31]. 

A two-component cleaning disinfectant concentrate dedicated for large surfaces, a ready-to-use (RTU) foam, and an RTU gel dedicated for local cleaning disinfection of low-touch surfaces or toilet bowls, respectively, were tested in presence of interfering substances in the quantitative suspension test according to EN 14476 [30] against polio-, adeno- and murine norovirus (MNV), in a quantitative carrier test with stainless steel disks without mechanical action according to EN 16777 [32], and in the four-field test also against adeno- and murine norovirus with mechanical action according to EN 16615 [33]. In line with the manufacturer’s recommendation, only the two-component cleaning disinfectant concentrate was tested at concentrations of 25, 50, and 100 ppm chlorine dioxide. Foam and gel preparations were tested undiluted.

## Materials and methods

### Preparation of suspensions of test strains

Virus suspensions were prepared by growing the viruses in cell lines producing high virus titers. Poliovirus type 1, LSc 2ab (NIBSC Collection Cat. No. 10/164) and adenovirus type 5 (ATCC Collection Cat. No. VR-5) strains were propagated in the HeLa cell line (ATCC Collection Cat. No. CCL-2). The MNV strain (Collection Cat. No. S99 Berlin) was propagated in the RAW 264.7 cell line (cat. No. ATCC TIB-71). Cell debris was centrifuged at 400 *g*_N_ for 15 minutes. The viral titers of the resulting suspensions were higher than 10^8^ TCID_50_/mL. This denoted the amount of virus (in mL) that would initiate a cytopathic effect in 50% of the cells [30].

### Tested products

- Product 1, *ARMEX 5 MD*, is a two-component cleaning disinfectant concentrate for large surfaces, which after dilution applied is at a concentrations of ClO_2_ of 25, 50, and 100 ppm, in combination with an activator consisting of acetic acid 25% with 20% surfactants.
- Product 2, *ARMEX 5 Foam*, is a ready-to-use foam used in combination with 1,500 ppm chlorine dioxide (0.15%) with citric acid 2.5% (activator) and 1% surfactant.
- Product 3, *ARMEX 5 WC*, is a ready-to-use gel in combination with 1,500 ppm chlorine dioxide with citric acid 2.5% (activator) and 1% surfactant.

The aqueous solution of activated chlorine dioxide for surface disinfection was obtained by mixing an adequate volume in hard water. The starting CIO_2_ concentration of 200 ppm in the solution was obtained by adding 50 mL of the precursor and 50 mL of the activator to 5 L of hard water (prepared according to EN 14476). The remaining lower concentrations were obtained by adequate dilution of the starting solution in hard water prepared according to EN 14476 [30].

### Determination of the chlorine dioxide concentration in the starting solution

The concentration of chlorine dioxide in the starting solution was determined on the basis of a direct measurement of the ultraviolet-visible spectroscopy (UV-VIS) spectrum in the range of 200 to 500 nm using a UV-VIS spectrophotometer (Hitachi U-2,900). Quantification is possible at λ_max_–358.5 nm, which is the absorption maximum for chlorine dioxide. The molar absorption coefficient of chlorine dioxide in the solution was determined using the least squares method [34], [35]. 

### Determination of virucidal activity in the suspension test

Quantitative suspension tests were carried out in accordance with EN 14476 [30]. The effectiveness of several concentrations of the two-component cleaning and disinfecting solution based on chlorine dioxide against polio-, murine noro-, and adenovirus was tested. First, 1.0 mL of the prepared virus suspension was added to 1.0 mL of 3.0 g/L albumin +3 mL/L sheep erythrocytes (interfering substances) and 8.0 mL of the test biocidal product. A virus control mixture was prepared using distilled water instead of the test product. After a contact time of 30 seconds and/or 5 minutes, virucidal activity was suppressed with 9 volumes of ice-cold preservative, Eagle minimal medium +2% fetal calf serum, and placed in an ice bath. Virus infectivity was determined using the endpoint titration method. For this purpose, a series of 10-fold dilutions of the test mixture were made and 0.1 mL of each dilution was transferred to six wells of a microtiter plate. Next, 0.1 mL of the host cell suspension with a density of more than 90% was added to allow monolayer formation. In parallel, a control assay was performed, where six wells did not contain virus suspension. The resulting cell samples were incubated at 37°C in 5% CO_2_ for 72 hours. The cells were examined for cytopathic effects (CPEs) using an inverted microscope. 

Viral titers were expressed as infectious dose for 50% of the cell culture (lg_10_ TCID_50_/mL). Calculations were made using the Spearman-Kärber method [36], [37]: It consists in evaluating the cytopathic effect of cell culture in prepared dilutions, starting with those dilutions where all cells have been infected, and ending with those where the virus does not multiply. The CPE was assessed in each of the six wells of the microtiter plate on a scale of 0 to 4, where 0 indicates no CPEs, 1=25% of cells show CPEs, and 4=100% of cells show CPEs. TCID_50_ was determined using Formulae 1 and Formulae 2.

Formulae 1: x=a–b 

Formulae 2: TCID_50_= 10^–x^, mL^–1^

Where *x* indicates a negative common logarithm of the 50% end point (–log_10_ TCID_50_), *a* is a negative common logarithm of the highest virus concentration used, *b* is a common logarithm of the dilution factor, and *c* is a sum of CPE percentages from all dilutions. 

The virucidal activity was determined as the difference between the TCID_50_/mL values in control and test samples. The result is expressed as reduction with a 95% confidence interval. A reduction in viral titer of 4 lg or higher (corresponding to an inactivation of ≥99.99%) is considered evidence of sufficient virucidal activity [30]. To check and control the entire study system, tests were also performed with the reference substance 0.7% glutaraldehyde solution.

### Determination of virucidal activity in the carrier test 

The quantitative carrier test according to the EN 16777 was performed in presence of interfering substances [32]. A total of 50 µL of the virus inoculum was deposited on each pretreated carrier and dried. Then, the inoculum was covered with 100 µL of test formulation (100 µL of hard water was applied as control) and incubated for 5 minutes. Immediately at the end of the exposure time, the disks were transferred into plastic vial holders with 900 µL of ice-cold culture medium to stop the activity of the formulation. Vials were vortexed for 1 minute to recover the residual viruses, and the eluate was immediately diluted 10-fold (quantal test method) to determine viral infectivity. Cytotoxicity was measured as described in the guideline [32]. In addition, a control of efficacy for suppression of the disinfectant’s activity was included.

### Determination of virucidal activity in the four-field test

The four-field test was performed, including the use of a specific organic load to simulate realistic environmental conditions that might affect the performance of disinfectants, according to EN 16615:2015 [33]. Briefly, four squares as test fields were marked on a coating material with a polyvinyl chloride (PVC) surface (20 cm×50 cm), making rows at a distance of 7 cm from one another. The marked test field 1 on this surface was inoculated with the inoculum based on the test virus suspension and the interfering substances defined in standard, namely the bovine albumin solution at 0.3 g/L, and to achieve high organic load conditions a bovine albumin solution at 3.0 g/L plus 3 mL of sheep erythrocytes. Here, 50 µL of inoculum were pipetted on the first test field (field 1) and spread with a glass spatula. Immediately after drying the inoculum on test field 1 at a temperature of 20°C to 25°C, the suitable test wipe (low-linting, non-woven 100% polypropylene) was fixed under a 2.3–2.5 kg of weight granite block. The weight defined above is intended to simulate the average pressure during the wiping process. For examination, the granite block with the fixed wipe was rapidly moved from test field 1 by hand exerting no additional force, to test field 4 and back for no longer than 2 seconds. At the end of the contact time (5 minutes chosen for all experiments), the test organisms were recovered from all four fields with a moistened and a dry cotton swab (producer: DELTALAB, Spain). The swabs of each field were transferred to 5 mL of minimum essential medium, and the tubes were vortexed for 60 seconds. Virus titers of the eluates were determined immediately by endpoint dilution techniques as described in EN 14476 [30] and calculated using the Spearman-Kärber method [36], [37]. The virus titer was expressed as lg TCID_50_/mL with a 95% confidence interval. The virus reduction was calculated by comparing the virus titers of each test field with those obtained immediately after drying and the chosen exposure time.

## Results

### Chlorine dioxide concentration in the starting solution

The coefficient for the precursor was 1290 L/mol * cm and was consistent with literature data [34], [35].

### Virucidal efficacy in quantitative suspension test in presence of interfering substances

**Figure 1:**
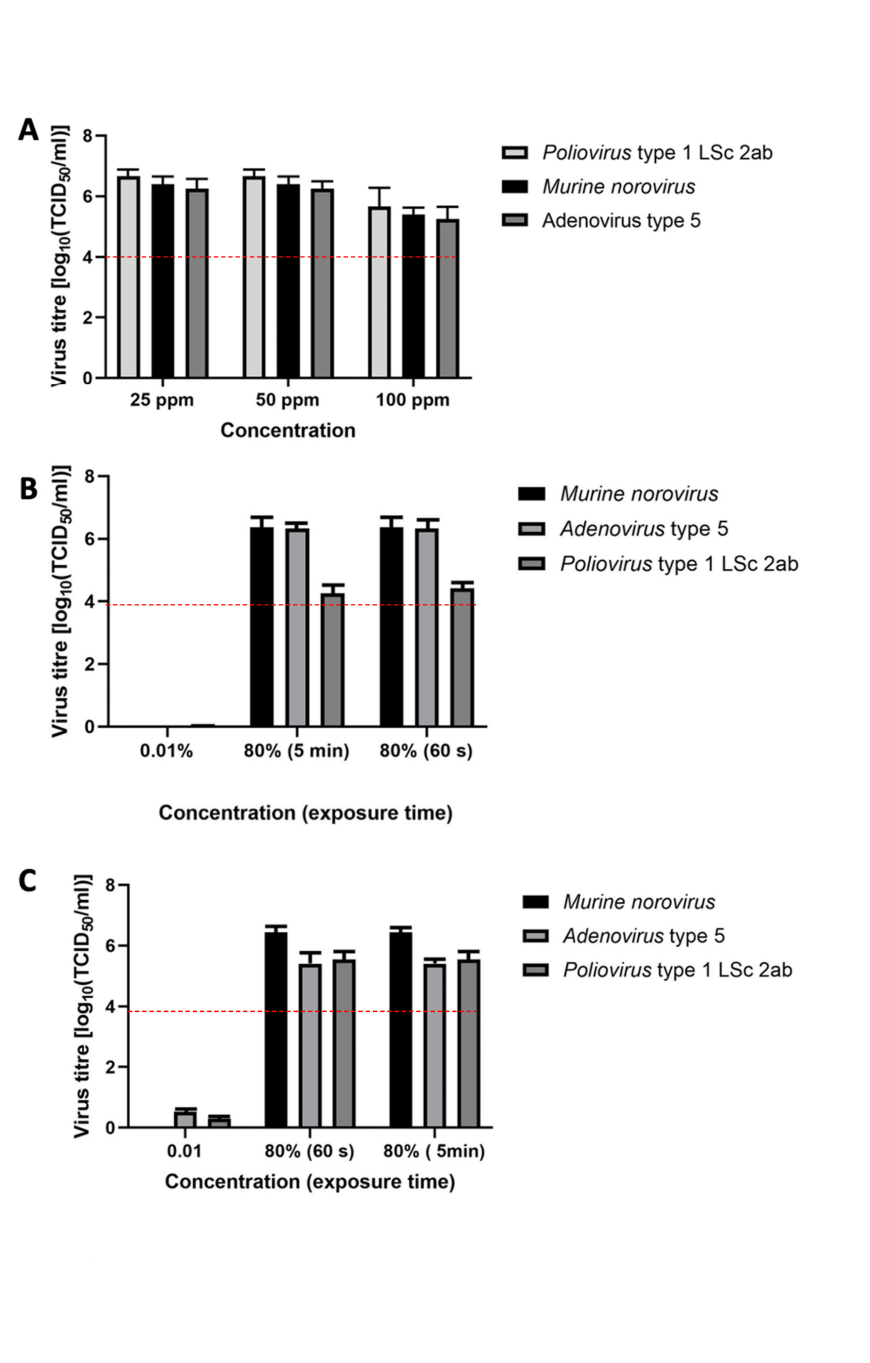
Virucidal efficacy in the quantitative suspension test in presence of interfering substances (A) product 1 at concentrations of 25, 50, and 100 ppm; (B) product 2; (C) product 3. Error bars show standard error; red line indicates a 4 lg reduction.

The tested viruses, including poliovirus, can be effectively inactivated. Figure 1A (Fig. 1) presents the virucidal activity of product 1. The mean reduction of poliovirus titers was ≤6.7 lg after 5 minutes of exposure at chlorine dioxide concentrations of 25 ppm and 50 ppm and ≤5.7 lg at a chlorine dioxide concentration of 100 ppm. The mean reduction of the murine norovirus titers was ≤6.4 lg after 5 minutes of exposure at chlorine dioxide concentrations of 25 ppm and 50 ppm and ≤5.4 lg at a chlorine dioxide concentration of 100 ppm. For adenovirus type 5, the mean titer reduction was ≤6.3 lg after 5 minutes of exposure at chlorine dioxide concentrations of 25 ppm and 50 ppm and ≤5.25 lg at a chlorine dioxide concentration of 100 ppm. 

Figure 1B (Fig. 1) presents the results of product 2. The mean reduction of the poliovirus titer was ≤4.4 lg after 60 seconds of exposure, that of the murine norovirus titer was ≤6.4 lg after 60 seconds of exposure, and the mean reduction of the adenovirus titer was ≤6.34 lg after 60 seconds of exposure. There was no further reduction of the three viruses after the exposure was extended to 5 minutes. In accordance with EN 14476 [30], one concentration (0.01%) was considered inactive.

Figure 1C (Fig. 1) presents the results of product 3. After 60 minutes of exposure, the mean reduction of the poliovirus titer was 5.54 lg, that of the murine norovirus titer in the suspension test was ≤6.4 lg, and the mean reduction of the adenovirus titer in the suspension test was ≤5.4 lg. There was no further reduction in the three viruses after the exposure was extended to 5 minutes. In accordance with EN 14476, one concentration (0.01%) was considered inactive.

### Virucidal efficacy in the carrier test in presence of interfering substances

**Figure 2:**
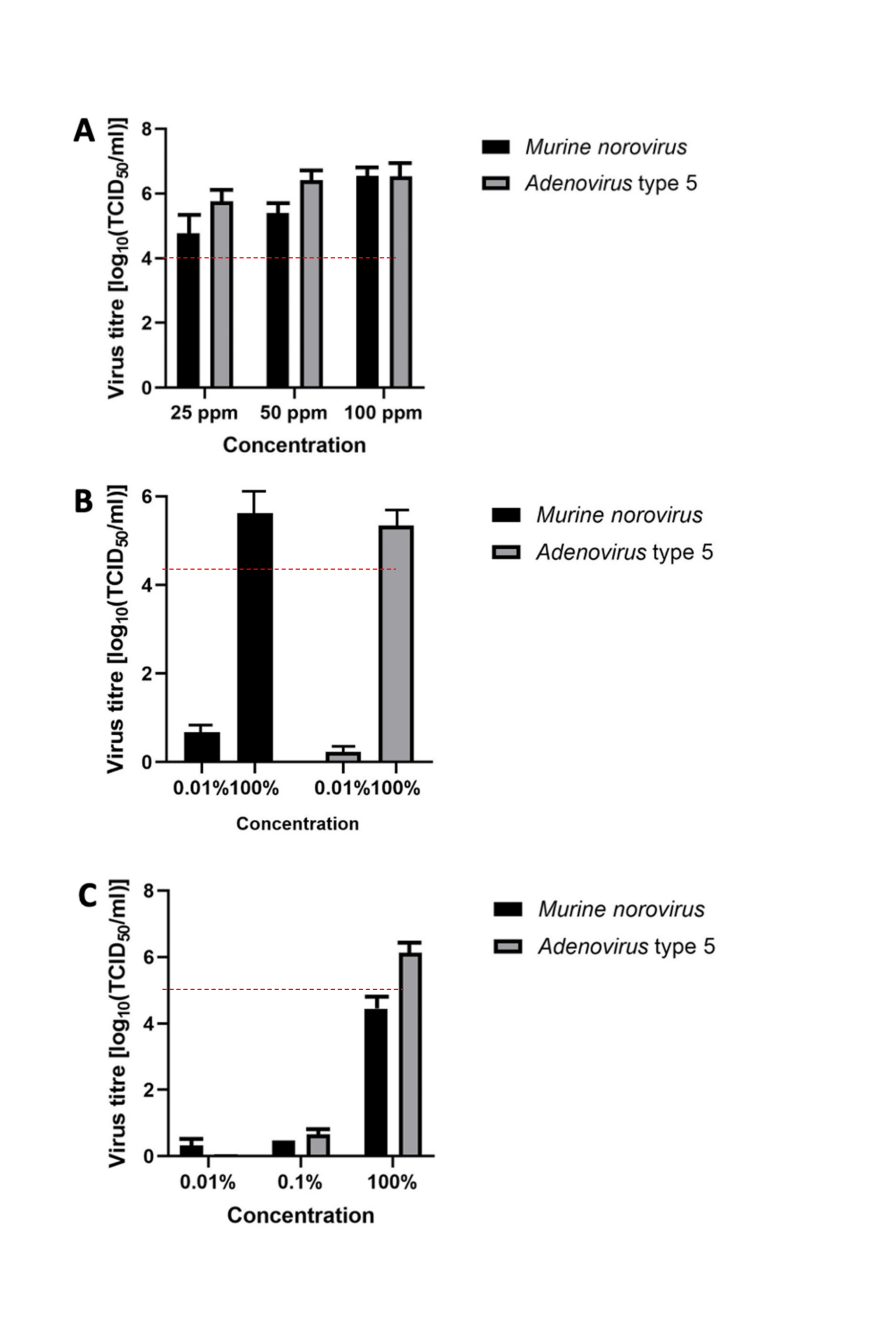
Virucidal efficacy in carrier test in presence of interfering substances (A) product 1 at concentrations of 25, 50, and 100 ppm; (B) product 2; (C) product 3; error bars show standard error. Red line indicates a 4 lg reduction.

Figure 2A (Fig. 2) presents the results of product 1. The mean reduction of the murine norovirus titer was ≤4.8 lg after 5 minutes of exposure at a chlorine dioxide concentration of 25 ppm. At concentrations of 50 ppm and 100 ppm, the mean reduction was ≤5.4 lg and ≤6.6 lg, respectively. In accordance with the EN 16777 [32], one concentration (0.01%) was considered inactive. For adenovirus, the mean titer reduction was ≤5.8 lg after 5 minutes of exposure at a chlorine dioxide concentration of 25 ppm. At concentrations of 50 ppm and 100 ppm, the mean reduction was ≤6.4 lg and ≤6.6 lg, respectively. In accordance with EN 16777 [32], one concentration (0.01%) was considered inactive.

Figure 2B (Fig. 2) presents the virucidal activity of product 2. The mean reduction of the murine norovirus titer was ≤5.6 lg after 5 minutes of exposure. The mean reduction of the adenovirus titer was ≤5.3 lg after 5 minutes of exposure. 

Figure 2C (Fig. 2) presents the virucidal activity of the RTU gel. The mean reduction of the murine norovirus titer was ≤4.44 lg after 5 minutes of exposure. The mean reduction of the adenovirus titer in the suspension test was ≤6.14 lg after 5 minutes of exposure. In line with EN 16777 [32], one concentration (0.01%) was considered inactive.

### Four-Field Test in presence of interfering substances

**Figure 3:**
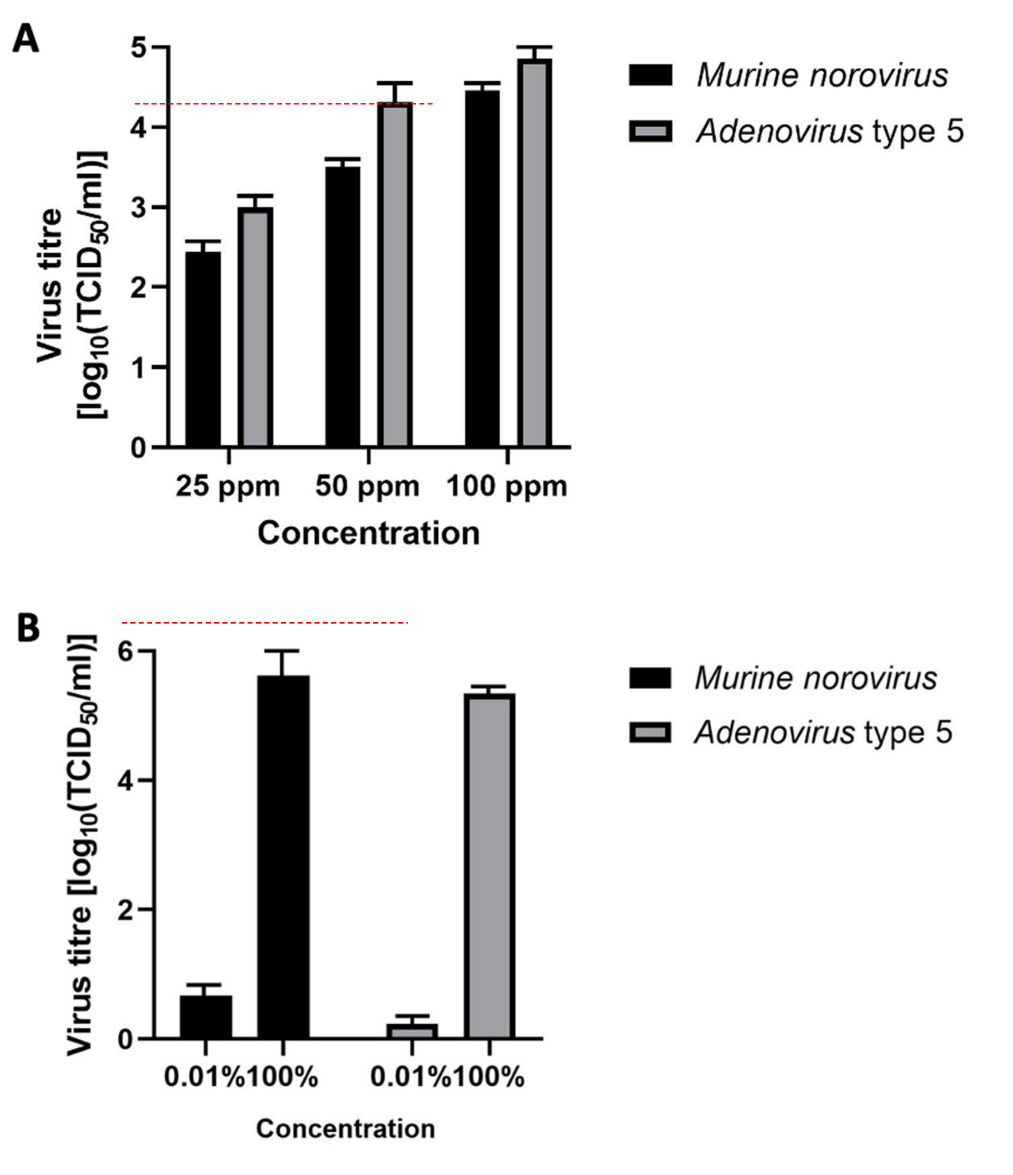
Virucidal efficacy in four-field test in presence of interfering substances; (A) product 1 at concentrations of 25, 50, and 100 ppm; (B) product 2; error bars show standard error. Red line indicates a 4 lg reduction.

Figure 3A (Fig. 3) presents the results of product 1. The mean reduction of the murine norovirus titer was ≤2.4 lg after 5 minutes of exposure at a chlorine dioxide concentration of 25 ppm. At concentrations of 50 ppm and 100 ppm, the mean reduction was ≤3.51 lg and ≤4.5 lg, respectively. The mean reduction of the adenovirus titer was ≤3.0 lg after 5 minutes of exposure at a chlorine dioxide concentration of 25 ppm. At concentrations of 50 ppm and 100 ppm, the mean reduction was ≤4.3 lg and ≤4.9 lg, respectively.

Figure 3B (Fig. 3) presents the results of product 2. The mean reduction of the murine norovirus titer was ≤6.2 lg after 5 minutes of exposure. The mean reduction of the adenovirus titer was ≤5.8 lg after 5 minutes of exposure.

**Figure 4:**
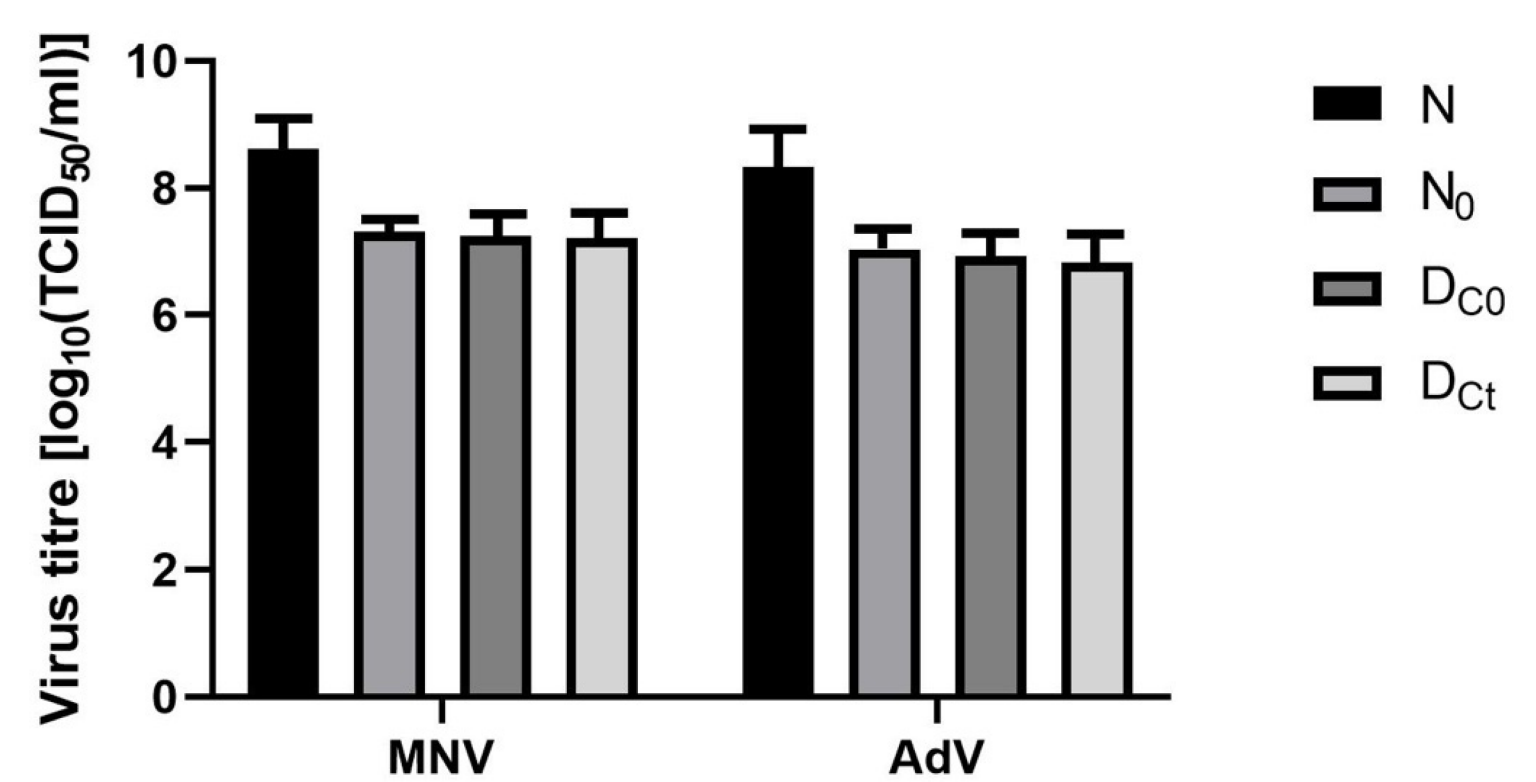
Stability of adenovirus type 5 and murine norovirus in presence of interfering substances (N_0_=virus titer applied to test surfaces, DC_0_=virus recovery immediately after drying, DC_t_=virus titer recovery after drying and contact time, N=virus titer in the test suspension). Error bars show standard error.

Initially, the virus titer was compared with the titers on the PVC carrier immediately after visibly drying and after 5 minutes of exposure. The calculated virus titer reductions after 5 minutes were 0.24 for the murine norovirus and 0.29 for the adenovirus (Figure 4 (Fig. 4)). 

## Discussion

### Method

The suspension test was conducted in accordance with EN 14476 [30] for the evaluation of virucidal activity of disinfectants or antiseptics that are homogeneous and physically stable when they are diluted with hard water. RTU products can only be tested at a concentration of 80% (97% with a modified method for special cases), because some dilution is always caused by adding the test organisms and appropriate interfering substances. As the criterion for virucidal activity, the standard assumes a reduction in the infectious titer of at least 4 lg [30], [32]. The disadvantage of suspension tests is the fact that the viruses are subjected to the activity of a large amount of disinfectant in suspension, which makes them more easily inactivated. Therefore, to assess the virucidal activity in practical conditions, it is necessary to use carrier tests. Thus, we conducted studies using the carrier test according to the EN 16777 [32]. In accordance with this, the reduction of murine norovirus and adenovirus titers was lower in the carrier test than in the suspension test. 

Because most surface cleaning and disinfection processes in medical facilities are done using mechanical action (mops, tissues, wipes), the virucidal activity was assessed additionally using the four-field test [33], which simulates practical conditions.

In the carrier tests as well as in the four-field test, only the murine norovirus and the adenovirus were used, because poliovirus is sensitive to drying on surfaces. 

### Results 

#### Quantitative suspension test

The lower reduction of polio-, murine noro-, and adenovirus titers at chlorine dioxide levels of 100 ppm vs 25 ppm and 50 ppm can be explained by viral aggregation, because aggregates are more resistant to disinfection. The aggregates can be linked to a drop in the pH of the working solution. Under such conditions, individual virions in the suspension can be induced to aggregate, as the pH of the solution drops due to the elimination of the repulsive electrostatic force (the isoelectric points of viruses are around 4.0) [38]. Virus aggregation is a natural process that helps viruses survive in the environment and increases their resistance to disinfection [39], [40], [41]. A similar phenomenon was observed for *Picornaviridae* [42]. However, this had no influence on meeting the criteria of EN 14476. 

The disadvantage of the suspension test is that the virus particles are suspended in a large volume of disinfectant, which facilitates the inactivation of viruses. As compared to the suspension tests, the ability of chlorine dioxide to inactivate viruses on stainless steel surfaces at the same concentration and exposure time was slightly lower for the solution and for the gel (Figure 3 (Fig. 3)). Reduced efficacy using the carrier method was confirmed by other authors [43]. For the foam, on the other hand, the carrier method did not decrease the reduction factor of the test viruses in relation to the suspension method (Figure 3 (Fig. 3)). Surface drying may limit the physical access of the disinfectant to virus particles [44]. Samandoulgou et al. [38] also showed that hydrophobic and van der Waals forces, as well as the isoelectric point and ionic strength, can promote the adhesion of nonenveloped viruses to solid surfaces, also reducing the access of the disinfectant to the virus. In most cases, dried virus, especially on the surface, is much more resistant to environmental influences than suspended particles. Therefore, to ensure that surface disinfectants are able to inactivate microorganisms, they must be tested for their effectiveness in near-real conditions, that is, in carrier tests, in which viruses are dried and deposited on the surface of a carrier simulating surfaces such as in EN 16777. Therefore, to ensure that surface disinfectants can inactivate the viruses, they must be tested in conditions that imitate real conditions, that is, in carrier tests where viruses are subjected to drying and deposited on the surface of the carrier, simulating such surfaces as those in EN 16777 [32]. In Poland, the first study of virucidal activity using carrier tests was conducted in 1965 [45]. 

The CEN characterized preparations intended for the disinfection of surfaces with and without mechanical action. In the assessment of virucidal activity, only the standard without mechanical action (i.e., EN 16777) is approved. In the future, the CEN plans to introduce a method involving mechanical action for the assessment of virucidal activity [46]. 

The selection of test viruses for a method with mechanical action was influenced by the existing carrier test without mechanical action (EN 16777). Viruses such as adenovirus and murine norovirus are also included in draft standards for assessing virucidal activity via suspension and carrier tests prEN 17914 [47] and prEN 17915 [48], respectively, in the following areas: surface disinfection in industries, food processing, distribution and retailing areas.

Choosing the most effective method of disinfectant application is crucial in the decontamination of surfaces contaminated by microorganisms, including viruses. In some studies, the use of a mechanical wiping factor was shown to increase viral load reduction [49], [50]. In our study, in the case of a product for cleaning and disinfecting large surfaces, it was necessary to increase the concentration of chlorine dioxide to 100 ppm against MVN in the four-field test with mechanical action within 5 minutes (Figure 3 (Fig. 3)). On the other hand, in the carrier test without mechanical action according to EN 16777, 25 ppm of chlorine dioxide was enough to achieve a 4 lg reduction (Figure2 (Fig. 2)). The reason for this phenomenon may be the amount of disinfectant. In the four-field test, the required volume of the solution is about 16 mL/m^2^ [51]. In the case of the carrier test without mechanical action, the required amount of the preparation is 318 mL/m^2^ [34]. The amount of agent applied to the disinfected surface is important. It was shown that amounts of preparation below 20 mL/m^2^ may not achieve an adequate disinfecting effect [52]. In addition, the type of carrier used may also influence the effectiveness of the preparation [53]. The PVC material used in the four-field method is the most difficult material to disinfect in the medical setting [53]. In the case of the RTU foam, the type of application was not significant, and the preparation had high virucidal activity in presence of interfering substances. This, in turn, can be associated with a concentration of the active substance greater than 100 ppm (Figure 2B (Fig. 2), Figure 3B (Fig. 3)). 

The methods for testing chemical disinfectants developed by the CEN take into account the different methods of applying the preparations to the surface (with and without mechanical action); additionally, they take into account the amount of preparation applied to the surface. In the case of EN 16777 without mechanical action, the lack of wiping is compensated for by an increased amount of disinfectant. It can be compared to the Sinner circle of optimal cleaning (mechanics-chemistry-time-temperature) used to compensate for individual factors affecting the optimization of cleaning and disinfection processes [54].

The duration of the preparation's action is an important element in the choice of the preparation. In practical terms, it should be as short as possible. According to European standards [55], the disinfection time for so-called touch surfaces is a maximum of 5 minutes for preparations intended for surfaces with bactericidal, fungicidal, tuberculocidal, and virucidal activity, and a maximum of 60 minutes for other surfaces. We tested all forms of chlorine-dioxide-based preparations for both touch surfaces and non-touch surfaces in a maximum of 5 minutes to ensure a realistic and feasible time of action of the preparations.

Most surface disinfection processes in medical facilities are carried out with mechanical action; therefore, it seemed reasonable to test according to the manufacturer’s instructions for cleaning using the four-field method with mechanical action. To our knowledge, there have been no studies evaluating the effectiveness of surfaced disinfectants in the three application forms tested here (concentrate, foam, and gel), assessed in suspension and carrier tests for virucidal activity, both with and without mechanical action. In addition, the four-field test specifies the amount of microorganisms transferred to the remaining test fields 2–4. Considering the lack of guidelines for viruses using the four-field standard, we found no virus transfer to other surfaces, which agrees with the findings of other authors [56]. This is due to the very low infectious doses of viruses; even a small amoung of virus transferred to uncontaminated areas may pose a risk of infection. In our research, no test virus transmission was observed when using either the foam or the concentrate. In the four-field test, MNV was the most stable virus (lg reduction after drying was 0.16), followed by adenovirus 0.27 (Figure 4 (Fig. 4)). An additional exposure time of 5 minutes resulted in only small decreases for MNV (0.24) and adenovirus (0.29) (Figure 4 (Fig. 4)). There was no transfer of test viruses to fields 2–4 when using any of the chlorine dioxide formulations.

The use of a mechanical factor in assessing the virucidal activity of wipes was also reflected in the ASTM E2967-15 standard [57]. Both four-field tests and ASTM E2967-15 have their limitations [58]. Nevertheless, they are very important for assessing the virucidal effect of chemical disinfectants in real-life conditions, including mechanical action.

## Limitations

Our work has several limitations. Only chlorine-dioxide-based formulations were tested. We used viruses that are specified in the standards, and we did not test on other surrogates or HuNoV. The Global Polio Eradication Program, which was initiated by the World Health Organization (WHO) in 1988 and outlined in the WHO global action plan to minimize poliovirus facility-associated risks after type-specific eradication of wild polioviruses, will require poliovirus to be replaced [59]. The parvovirus minute virus of mice might be a candidate, but it was not tested in our research.

## Conclusions

Based on the results, we indicated that cleaning and disinfecting preparations based on chlorine dioxide at a concentration of 25 ppm chlorine dioxide are active against polio-, adeno-, and norovirus for 5 minutes in presence of interfering substances in the suspension and carrier tests, in accordance with European standards EN 14476, EN 16777, and in the four-field test according to EN 16615. The European Standards are reliable and repeatable, and allow the user to compare products.

## Notes

### Competing interests

Authors AC and WH are employees of MEXEO Institute of Technology, Kedzierzyn-Kozle, Poland.

The other authors declare that they have no competing interests.

### Authors’ ORCID

- Patryk Tarka: 0000-0003-1421-4563
- Arkadiusz Chrusciel: 0000-0001-9918-4134
- Wieslaw Hreczuch: 0000-0002-0435-7686
- Krzysztof Kanecki: 0000-0001-8931-8565
- Aneta Nitsch-Osuch: 0000-0002-2622-7348

### Funding

No. POIR.01.01.01-00-1104/17-00 *“Technology for the manufacture and use of chlorine dioxide-based disinfectant formulations to combat epidemic outbreaks of pathogenic microorganisms with high resistance to chemical disinfection”*, implemented under the Operational Program Intelligent Development 2014-2020 co-financed by the European Regional Development Fund.

### Data availability statement

The data that support the findings of this study are available on request from the corresponding author.

### Acknowledgments

The authors thank EKOLABOS, Wroclaw, Poland, for technical support and materials used for the experiments.
